# Sox9 Duplications Are a Relevant Cause of Sry-Negative XX Sex Reversal Dogs

**DOI:** 10.1371/journal.pone.0101244

**Published:** 2014-07-10

**Authors:** Elena Rossi, Orietta Radi, Lisa De Lorenzi, Annalisa Vetro, Debora Groppetti, Enrico Bigliardi, Gaia Cecilia Luvoni, Ada Rota, Giovanna Camerino, Orsetta Zuffardi, Pietro Parma

**Affiliations:** 1 Department of Molecular Medicine, Pavia University, Pavia, Italy; 2 Department of Agricultural and Environmental Sciences, Milan University, Milan, Italy; 3 Biotechnology Research Laboratories, Fondazione IRCCS Policlinico San Matteo, Pavia, Italy; 4 Department of Veterinary Science and Public Health, Milan University, Milan, Italy; 5 Department of Veterinary Science, Parma University, Parma, Italy; 6 Department of Health, Animal Science and Food Safety, Milan University, Milan, Italy; 7 Department of Veterinary Science, Torino University, Torino, Italy; Baylor College of Medicine, United States of America

## Abstract

Sexual development in mammals is based on a complicated and delicate network of genes and hormones that have to collaborate in a precise manner. The dark side of this pathway is represented by pathological conditions, wherein sexual development does not occur properly either in the XX and the XY background. Among them a conundrum is represented by the XX individuals with at least a partial testis differentiation even in absence of SRY. This particular condition is present in various mammals including the dog. Seven dogs characterized by XX karyotype, absence of SRY gene, and testicular tissue development were analysed by Array-CGH. In two cases the array-CGH analysis detected an interstitial heterozygous duplication of chromosome 9. The duplication contained the *SOX9* coding region. In this work we provide for the first time a causative mutation for the XXSR condition in the dog. Moreover this report supports the idea that the dog represents a good animal model for the study of XXSR condition caused by abnormalities in the SOX9 locus.

## Introduction

Gonadal differentiation in mammals is initiated, controlled, and regulated by the coordinated action of several genes and hormones.

During the last two decades many genes involved in this process have been identified [1], and in recent times, epigenetic factors have also come into play [2].

The scepter of power remains firmly in the hands of *SRY,* the sex determination key gene [3] located on the Y chromosome that is necessary and sufficient to induce the primordial undifferentiated gonad to develop into a testis [4]. In the absence of *SRY*, that is in the XX embryos a different set of genes is activated, and the undifferentiated gonad becomes an ovary [5]. *SRY* role takes place in a short period of timeand ceases after the activation of *SOX9*. This gene is a main actor in testis differentiation and in several other embryogenetic fields [6]. Normally this process follows well-defined tracks: the XY embryos develop the testis and a male phenotype, while the XX embryos develop ovaries and a female phenotype. However, this complex process can result in the appearance of developmental errors on account of the discordance between the chromosomal, gonadic, and phenotypic sex.

One of the most interesting issues is represented by the XX sex-reversal cases. In humans most of them do have the SRY gene that is transposed to the tip of Xp due a recurrent Non Allelic Homologous Recombination between of PRKX and PRKY in a particular Y haplotypic background [7]. However, in both humans and other mammals SRY-negative XX males have been observed displaying testicular tissue, with or without ovarian tissue. It has been observed, at least in pig, that in these cases *SOX9* gene is surprisingly activated in the absence of *SRY* [8]. Subjects with XX sex reversal have been observed in different species: human, pig, goat, llama, dog, and horse [9]–[14]. In the dog this pathology appears with a relatively high frequency compared to the other species and has been described in various breeds [15]. XX sex-reversal in dogs can show a very different structure of gonads, ranging from bilateral testis to one ovo-testis and one ovary. With regard to the causes of occurrence of this anomaly in different species, to date, only three genetic causes have been identified: *FOXL2* in goat [16] and *SOX9* [17] and RSPO1 [18] in humans. *SOX9* alterations in XXSR cases include duplications, triplications, and reciprocal translocations [17], [19]–[21]. Surprisingly, despite the many cases investigated in XXSR dogs, till date no causative mutations have been reported, but only a linkage for a genomic region has been detected in a single specific pedigree [22].

In this article, we report the molecular analysis of seven XX sex-reversal dogs and we clearly show, for the first time in literature, that two of them carry *SOX9* gene duplication.

## Materials and Methods

### Case Description

Seven dogs from different breeds have been considered in this study: Four of these have already been described, while three are still unreported. Case C2, C9, C10, and C44 [23]–[25] have been previously characterized to show a presence of testicular tissue with a XX karyotype in the absence of the *SRY* gene. The other three cases, C61, C64, and C65, have been characterized in this study.

### Histological Examination

All clinical activities and surgical experiments on the dogs were carried out at the Veterinary Hospital of the University of Milan by veterinary surgeons. During the research no animals were sacrificed. The anesthetic and surgical protocol fulfilled the Federation of European Laboratory Animal Science Association’s recommendations and European Union legislation (Council Directive 86/609/EEC). Blood (1.5 ml) and gonad samples were collected for a routine medical procedure and stored for further analysis. Consistent with Italian regulation (D.L. 116/1992), the owners signed a voluntary consent, for their animals before undergoing surgery. This consent includes the possibility that the removed tissue may be used in scientific researches without economic interest.

After surgical excision of the gonads, they were fixed in 10% neutral buffered formalin for at least three days. For histological examination, several slices of gonads were processed histotechnologically according to standard laboratory procedures, cut at 5 µm, and stained with hematoxylin and eosin [26].

### Cell Cultures and Genetic Analyses

Peripheral blood lymphocyte cultures were performed following the standard procedures [27]. *SRY* gene analysis was performed as reported [23]. Briefly, the entire SRY coding region (GenBank AF107021) was amplified by polymerase chain reaction (PCR) using the following primers: (5′-3′): SRY-Dog-F: ctttccaacttccctccgta and SRY-Dog-R: ggacgtttcgttagccagag. The PCR product was 813 bp long. PCR was performed using AmpliTaq Gold DNA Polymerase (Applied Biosystems) according to the manufacturer’s instructions.

### Array-CGH Analyses

Array-CGH was performed using a custom Agilent Canine Genome CGH Microarray 180 K (Agilent Technologies, Santa Clara, California, USA) and processed as reported [28]. Briefly, 500 ng of purified DNA of a subject and a control, were double-digested with RsaI and AluI for two hours at 37°C. After 20 minutes at 65°C, each digested sample was labeled by the Agilent random primers; labeling was performed for two hours using Cy5-dUTP for the subject DNA and Cy3-dUTP for the control DNA. The labeled products were columns purified and prepared according to the Agilent protocol. After probe denaturation and pre-annealing with 5 µl of Cot-1 DNA, hybridization was performed at 65°C, with rotation for 40 hours. After two washing steps, the arrays were analyzed with the Agilent scanner and the Feature Extraction software (v10.7.3.1). A graphical overview was obtained using the CGH analytics software (v7.0.4.0). The DNA extracted from a normal female (boxer breed) was used as the control in all cases. All experimental data were submitted to GEO repository with the following Series accession number: GSE57137.

### Quantitative real Time PCR

*Sox9* duplications detected by array CGH were confirmed by Real-Time-qPCR with SYBR Green detection (Brilliant II SYBR Green QPCR master mix, Agilent Technologies), using one non-polymorphic marker located within the duplicated region. The primers were designed by using the Primer3 Software online (http://frodo.wi.mit.edu/primer3/), with the following criteria: Amplicon size 80–200 bp, GC content of 20–80%, and melting temperature (Tm) of 59–61°C. The primer sequences are available on request. Real-time detection was performed using the Stratagene Mx3000P. The Real-Time-qPCRs were performed in triplicate for each reaction.

The comparative CT method (ΔΔCT method) was used to discriminate between two and three allele copies of the DNA target sequence (Sox9) in the two dogs (resulted duplicated by a-CGH) relative to five normal control dogs DNA samples. The data have been normalized against two different reference sequences (Abs17, Bglr2).

## Results

**Figure 1:**
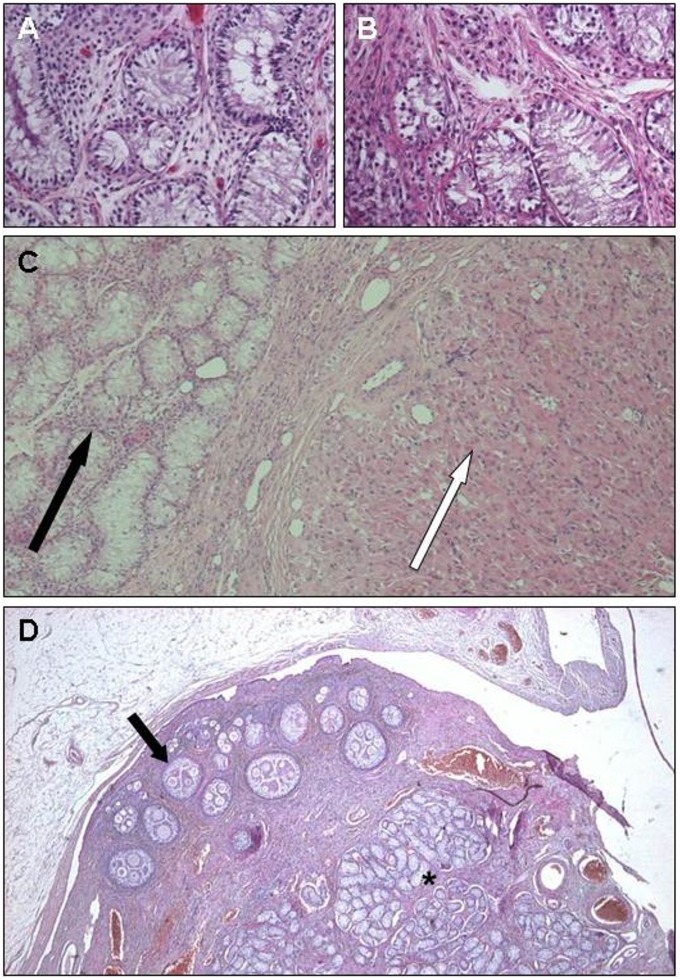
Histological examination of the new cases reported. Case C61: Histologic section of the right (A) and left (B) gonad showing seminiferous tubules with diffuse atrophy of the seminal line. Case C64: Right Ovotestis (C): The gonads were surrounded by ovarian bursa and shown some follicular structures and corpora lutea (white arrow). In the medulla hypoplastic seminiferous tubules were present (black arrow). Case C65 (D): Dog ovotestis. In the gonad, follicular structures including oocytes (arrow) coexist with testicular tubuli lined by Setoli cells (asterisc) (Courtesy of Valeria Grieco, University of Milan).

All the three new cases, C61, C64, and C65, showed a normal 78,XX karyotype in all the observed metaphases, and PCR analyses confirmed the absence of the *SRY* gene (not shown). Moreover, the histological analyses revealed the presence of testicular tissue in all the three cases, indicating that the male pathway was active during the fetal period in the absence of *SRY.* The testes of Case 61 are composed of testicular parenchyma with absence of the germline. In Cases 64 and 65 the right and left gonads are ovotestes (Figure 1a, b, c, d).

The results of array-CGH in the seven cases analyzed are listed in Table 1.

**Table 1:** List of CNVs identified with array-CGH in the seven cases with the indication of their code, type, location and size (CanFam2 assembly).

Casecode	CFA	DEL/DUP	CNVcode	Logratio	Size Kb	Lastunaffectedbp	Firstaffectedbp	Lastaffectedbp	Firstunaffectedbp	AlreadyDescribed(Y/N)	Genes
C2	9	DEL	1	−0.7	459	20,436,097	20,465,561	20,924,123		Y	
	9	DUP	2	0.5	541		21,021,894	21,562,129	21,574,304	Y	
C9	9	DEL	1	−0.8	459	20,439,097	20,465,561	20,924,123		Y	
	9	DUP	2	0.5	541		21,021,894	21,562,129	21,574,304	Y	
C10	9	DUP	3	0.5	577	10,414,955	11,016,965	11,593,933	12,062,144	N	SOX9
	9	DUP	4	0.3	414	19,864,938	20,022,338	20,436,297	20,447,061	Y	
	9	DEL	1	−0.75	458	20,447,061	20,465,561	20,924,123		Y	
	9	DUP	2	0.5	541		21,021,894	21,574,304	21,589,624	Y	
C44	9	DUP	3	0.57	577	10,414,955	11,016,965	11,593,933	12,062,144	N	SOX9
	9	DEL	5	−0.9	1300	19,766,692	19,819,256	21,119,179	21,292,889	Y	
C61	9	DEL	6	−0.8	809	20,097,414	20,115,306	20,924,123		Y	
	9	DUP	2	0.5	541		21,021,894	21,562,129	21,574,304	Y	
C64	9	DEL	6	−0.8	809	20,097,414	20,115,306	20,924,123		Y	
	9	DUP	2	0.5	541		21,021,894	21,562,129	21,574,304	Y	
C65	9	DEL	6	−0.8	809	20,097,414	20,115,306	20,924,123		Y	
	9	DUP	2	0.5	541		21,021,894	21,562,129	21,574,304	Y	

CNVs were checked for occurrence in the Database of Genomic Copy Number Variants in the dog genome (http://dogs.genouest.org/LUPA.dir/CNV.html) and in several papers [29]–[33].

**Figure 2:**
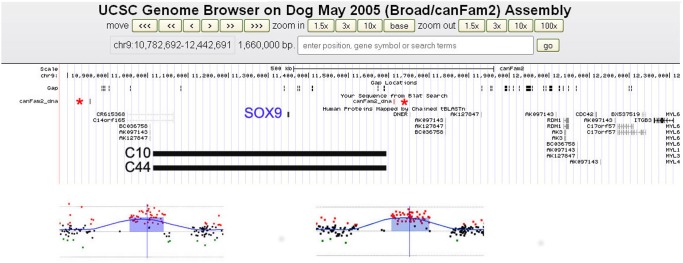
Graphical representation of the SOX9 locus duplications discovered. The figure shows a 1,6(canFam2 assembly) and magnified views of the two SOX9 duplications detected, by array-CGH, in cases C10 (left) and C44 (right), respectively. The shaded areas indicate a gain in DNA copy number (duplication, average log2 ratios: +0, 5) detected by red dots. Asterisks indicate the 168 bp repeats.

**Figure 3:**
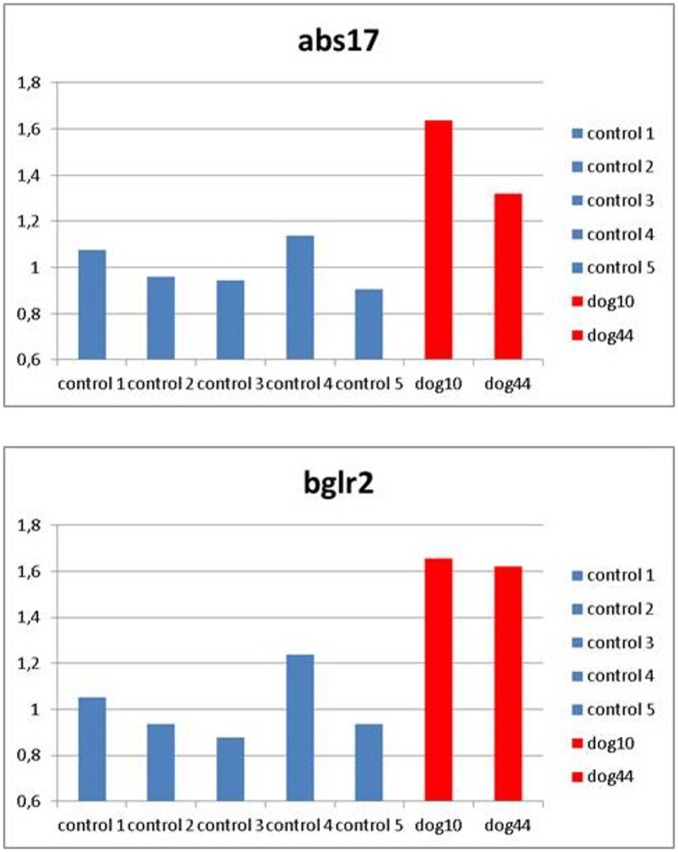
Q-RT PCR results. Histograms represent the copy numbers ratio of a non-polymorphic probe within Sox9 gene in the two duplicated dogs (dog10 and dog44, red bars) relative to five normal control dogs (blue bars). The data have been normalized against two different reference sequences (Abs17, Bglr2).

In cases C10 and C44, the array-CGH analysis detected an interstitial heterozygous duplication of chromosome 9, of 577 Kb (from 11,016,965 to 11,593,933; all data are referred to CanFam2 genome assembly) (Figure 2). The duplication contained the *SOX9* gene. This was confirmed by *reverse transcriptase* (RT)-PCR (Figure 3) and it was never described as c*opy number variation*s (CNVs) in different dog breeds [29]–[33]. The duplicated region was flanked by small, 168 bp, directly oriented repeats of >97,6% sequence identity, suggesting that non-allelic homologous recombination (NAHR) might have mediated these duplications. Furthermore, array-CGH identified several CNVs (data not shown) in our cases; all of them were described as polymorphic in previous dog aCGH reports [29]–[33]. The complex CNV region on CFA9∶19,761,852–21,600,512 has been observed, and reported with slightly different boundaries depending on the array platform used, in multiple studies [29]–[33]. Several CNV patterns have been described: gains or losses across the whole region, gains or losses of only a part of the region or alternate gains and losses within a single individual. The desert region between 20,115,306 and 21,119,179 is orthologous (61.9% of bases, 84.0% of span; http://genome.ucsc.edu/index.html) to the human region chr17∶68,723,331–69,717,418 (genome assembly Hg19), located 500–600 Kb upstream of SOX9, which is suggested to be the human regulatory region critical for gonadal SOX9 expression [20]. It is particularly interesting because, taking into account that the Dog Genome Assembly is a working progress and contains many assembly errors (Rossi E. personal communication), the actual distance between SOX9 and this region within the dog genome could be the same of the human one. A more stable and defined dog assembly will demonstrate the actual distance between the two regions and will help to clarify the related effects. As shown in Table 1, in our cases the CNV from CFA9∶19,761,852–21,600,512 has different patterns: complex in cases 2, 9, 10, 61, 64, 65 and simple as a deletion in case 44.

## Discussion

Genes in the SOX family play a critical role in the sex determination process. *SRY* is the master gene of this process [34] while *SOX9* represents the genetic factor that, activated by *SRY*, starts and regulates testis development. Although *SRY* is mammal-specific (with very few exceptions) *SOX9* plays an important role in bird also [35]. *SOX3* and *SOX8* genes are also involved in the sex determination process [36]–[37]. Chromosomal duplications as well as triplications involving the *SOX9* locus on HSA17q24.3, have been reported to be the causative mutations of the XX sex-reversal condition [38], however, all these duplications/triplications, except one, do not involve the *SOX9* coding region (CDS), but all are located 5′ to this gene. Indeed only the first reported *SOX9* duplication includes the SOX9 CDS [39]. This duplication, characterized by *Variable-Number Tandem Repeat* (VNTR) analyses, is at least 11.7 Mb long and is starts at 9.4 Mb 5′ and ends at 2.2 Mb 3′ of SOX9 CDS.

The mechanism underlying the XXSR condition in the presence of SOX9 duplications is still not clear, although it is clear that a *Sox9* over expression is required to induce the testis development in a XX background.

Therefore all different Sox9 locus duplications must be organized to allow this possibility. The Sox9 transgenic mouse effectively develops the XXSR phenotype. Incidentally, in this case, the gene is under the regulation of a strong promoter, which is able to activate the Sox9 expression in the right place at the right time, also in the absence of *Sry* [40]. *SOX9* is initially expressed in both the developing gonads (XY and XX), but only in the XY gonads its expression increases greatly. This upward regulation is due to *SRY* activation, and later on, to an auto-loop reinforced by additional positive feed-forward signals (Fgf9). In the XX developing gonad the auto-loop is not able by itself to up-regulate *Sox9* expression; moreover, female-specific genes repress additional feed-forward signals.

The analyses of human duplications in XXSR suggest a model of action. In these subjects two CDS SOX9 doses are present (as in normal subject), but the upstream region in one allele is duplicated, and this condition probably induces Sox9 over-expression.

The perturbation of sex determination process may be caused either by gain of function (GOF) or Loss of function (LOF) mechanisms. In the first case male gene (i.e. SOX9 in human and probably in pig) are involved whereas in the second ones female genes are involved (i.e. RSPO1 in human and FOXL2 in goat). In addition, the mechanisms of LOF are often associated with more severe phenotypes that include other abnormalities. For this reason we believe that SOX9 GOF remains the most reasonable candidate mechanism to explain the remaining unexplained XXSR cases in the involved species.

The dog represents a good animal model for the study of this disease because it shows relative high frequencies of XXSR cases and more precisely it can be a valuable model for the study of XXSR cases caused by SOX9 locus duplication.

In addition, the dog could represent an alternative animal model to the mouse considering that it seems to be different from other mammals at least for: a) SRY expression [41]; b) role of TESCO genomic region [42] and gene-dosage sensibility [43].

Unfortunately the genome assembly around the *SOX9* gene in the dog (CanFam3) seems to possess many assembly problems and consequently comparative analyses between this locus and the homolog locus in other species is quite difficult (Rossi E. personal communication).
